# Hypoxia Regulates the Self-Renewal of Endometrial Mesenchymal Stromal/Stem-like Cells via Notch Signaling

**DOI:** 10.3390/ijms23094613

**Published:** 2022-04-21

**Authors:** Sisi Zhang, Rachel W.S. Chan, Ernest H.Y. Ng, William S.B. Yeung

**Affiliations:** 1 Department of Obstetrics and Gynaecology, LKS Faculty of Medicine, The University of Hong Kong, Hong Kong 999077, China; u3006008@connect.hku.hk (S.Z.); nghye@hku.hk (E.H.Y.N.); 2 Shenzhen Key Laboratory of Fertility Regulation, The University of Hong Kong Shenzhen Hospital, Shenzhen 518000, China

**Keywords:** endometrial mesenchymal stromal/stem cells, label-retaining stromal cells, hypoxia, Notch signaling, HIF-1α

## Abstract

Human endometrium is an incredibly dynamic tissue undergoing cyclic regeneration and shedding during a woman’s reproductive life. Endometrial mesenchymal stromal/stem-like cells (eMSC) contribute to this process. A hypoxic niche with low oxygen levels has been reported in multiple somatic stem cell types. However, the knowledge of hypoxia on eMSC remains limited. In mice, stromal stem/progenitor cells can be identified by the label-retaining technique. We examined the relationship between the label-retaining stromal cells (LRSC) and hypoxia during tissue breakdown in a mouse model of simulated menses. Our results demonstrated that LRSC resided in a hypoxic microenvironment during endometrial breakdown and early repair. Immunofluorescence staining revealed that the hypoxic-located LRSC underwent proliferation and was highly colocalized with Notch1. In vitro studies illustrated that hypoxia activated Notch signaling in eMSC, leading to enhanced self-renewal, clonogenicity and proliferation of cells. More importantly, HIF-1α played an essential role in the hypoxia-mediated maintenance of eMSC through the activation of Notch signaling. In conclusion, our findings show that some endometrial stem/progenitor cells reside in a hypoxic niche during menstruation, and hypoxia can regulate the self-renewal activity of eMSC via Notch signaling.

## 1. Introduction

The stem cell niches refer to the specific local microenvironments, including cellular and acellular components, that integrate both systemic and local cues to modulate the biology of stem cells [1,2]. Emerging evidence suggests that oxygen levels are usually much lower in the stem cell niches than those in the ambient air or even in the host tissues [3,4]. For instance, the oxygen levels in the niches of hematopoietic, mesenchymal and neural stem cells are 1–6%, 2–8% and 1–8%, respectively [5,6,7]. It has been widely documented that low oxygen tension (hypoxia) plays a critical role in the maintenance of stem cells, and the hypoxic niches tightly regulate the self-renewal and differentiation of the resident stem cells [3,8,9].

Human endometrium undergoes cyclical breakdown and regeneration under the regulation of progesterone and estrogen [10]. Recent studies suggest that a rare population of endometrial mesenchymal stem cells (eMSC) contributes to this extensive cellular turnover after menstruation [11]. The co-expression of two perivascular markers: CD140b and CD146 can be used to purify eMSC [12]. The possibility that hypoxia plays a physiological role at menses has been proposed for over 70 years [13]. The presence of hypoxia, usually defined as a partial oxygen pressure below 10 mmHg, can be detected by pimonidazole staining. In a hypoxic area, pimonidazole forms stable protein adducts which can be visualized by the specific antibody [14]. Since then, many scientists have explored the role of hypoxia in the endometrium, with conflicting results from in vitro, ex vivo and in vivo models [15,16,17]. Until now, there have been very few studies investigating the relationship between hypoxia and the endometrial stem/progenitor cell during endometrial breakdown. It remains largely unknown how hypoxia actively regulates the behavior of eMSC. To gain insight into the presence of endometrial hypoxia and its role on endometrial stem/progenitor cells, we utilized the label-retaining cells approach to identify endometrial stem/progenitor cells in a mouse menstrual-like model. Given that stem cells divide infrequently, they can retain the DNA synthesis label (bromodeoxyuridine (BrdU)) after a prolonged chase period. The BrdU^+^ cells after a 6-week chase were referred as LRSC in the present study [18]. Mice, along with other mammalian species whose endometria do not undergo spontaneous decidualization, do not normally menstruate. Nevertheless, menstruation can be stimulated in mice using a well-established protocol to recapitulate the events that occur in the human endometrium at menses [19].

Notch signaling is a highly conserved signaling pathway, and is crucial for cell fate specification and the maintenance of somatic stem cells [20]. The Notch signaling is frequently activated under hypoxic conditions to modulate stem cell properties [21,22]. In neural stem cells, HIF-1α synergizes with the Notch intracellular domain (NICD) to prevent the differentiation of neural stem cells [23]. Integration between hypoxia and Notch signaling has also been described in embryonic stem cells [24]. Whether HIF-1α modulates Notch signaling in eMSC under hypoxic conditions remains to be investigated.

In the current study, we determined whether a hypoxia episode occurred in endometrial repair during menstruation in a mouse menstrual-like model. We found that the LRSC were located in a hypoxic microenvironment when the mouse endometrium underwent intensive tissue remodeling. A hypoxic condition significantly enhanced the self-renewal capacity, colony-forming ability and proliferation of eMSC in vitro. The observed effects were mediated via a HIF-1α/Notch signaling pathway.

## 2. Results

### 2.1. LRSC Reside in A Hypoxic Microenvironment during Endometrial Breakdown and Early Repair

**Figure 1:**
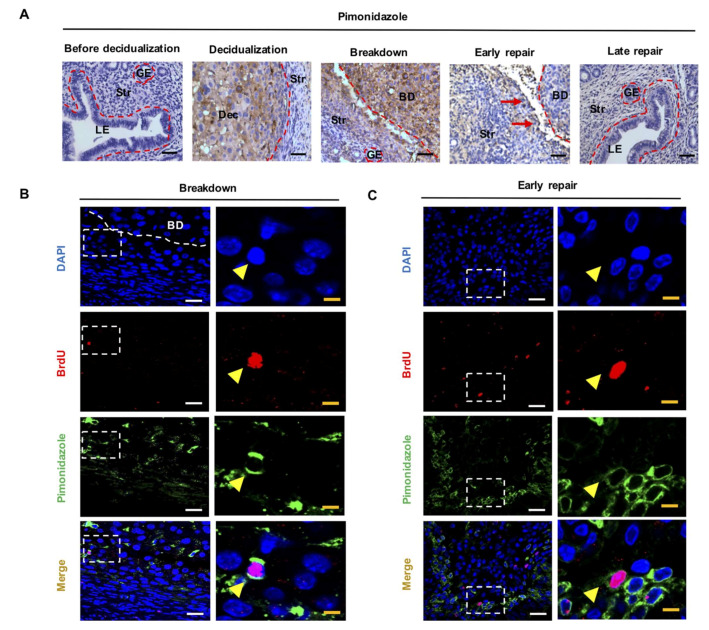
LRSC reside in a hypoxic microenvironment during endometrial breakdown and early repair. (**A**) Immunohistochemical images of pimonidazole staining (brown) at different time points of the mouse menstrual-like model, scale bar: 20 µm. Red arrows indicate the unrepaired area. Representative immunofluorescence images show LRSC colocalizing with hypoxic marker pimonidazole (yellow arrow) at (**B**) breakdown and (**C**) early repair. Right panels are enlarged figures of white box in the left panels. Scale bars: left panels 20 µm; right panels 5 µm. *n* = 3–5 mice per group. Abbreviations: BD, breakdown; BrdU, bromodeoxyuridine; Dec, decidua; GE, glandular epithelium; LE, luminal epithelium; LRSC, label-retaining stromal cells; Myo, myometrium; Str, stroma.

Pimonidazole is a marker of tissue hypoxia and can only be detected in areas where oxygenation is lower than 10 mmHg [25]. In hypoxic cells, it forms stable adducts with peptides and proteins at an amount proportional to the level of hypoxia [26]. Hypoxia was not detected in the mouse endometrium before decidualization (Figure 1A). During decidualization, pimonidazole staining was intense and limited to the decidualized zone. At the time of endometrial breakdown, the hypoxic region was localized to the decidualized and basal stroma. During early repair, the pimonidazole staining was only detected at the margins of unrepaired tissues. The pimonidazole staining gradually diminished as the endometrium restored (Figure 1A). The results demonstrated a transient but intense hypoxic episode in the mouse endometrium of simulated menstruation.

**Figure 2:**
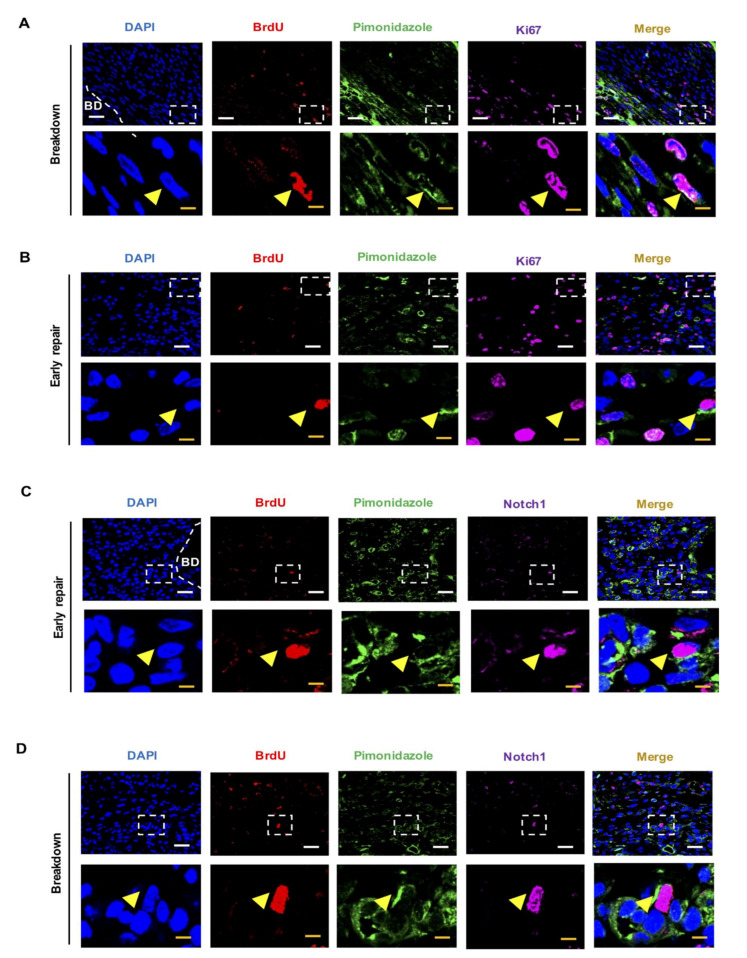
Characterization of LRSC localized in the hypoxic microenvironment during breakdown and early repair. Representative immunofluorescence images showing colocalization of the hypoxic LRSC with the proliferating marker Ki67 (yellow arrow) at (**A**) breakdown and (**B**) early repair. Hypoxic LRSC colocalizing with Notch1 (yellow arrow) at (**C**) breakdown and (**D**) early repair. Lower panels are enlarged figures of white box in the upper panels. Scale bar: upper panel 20 µm; low panel 5 µm. *n* = 3–5 mice per group. Abbreviations: BrdU, bromodeoxyuridine; BD, breakdown; LRSC, label-retaining stromal cells.

Next, we evaluated the colocalization of LRSC and pimonidazole staining at breakdown and early repair, since hypoxia was detected at these timepoints. LRSC were experienced in a hypoxic microenvironment during breakdown and early repair; 9.56 ± 1.74% (Figure 1B, *n* = 3) and 10.46 ± 4.42% (Figure 1C, *n* = 5) of the LRSC were positive for pimonidazole staining, respectively. Further investigation demonstrated that 21.43 ± 5.68% of the hypoxic LRSC underwent proliferation at breakdown (Figure 2A, *n* = 3), and increased to 50 ± 10.68% by early repair (Figure 2B, *n* = 3). Surprisingly, about half of the hypoxic LRSC colocalized with Notch1 immunoreactivities (breakdown: 52.38 ± 4.12%, Figure 2C, *n* = 3; early repair: 53.81 ± 16.06%, Figure 2D, *n* = 3), indicating the Notch signaling pathway was activated in these stem/progenitor cells.

### 2.2. Hypoxia Facilitates the Proliferation and Self-Renewal Activities of eMSC

**Figure 3:**
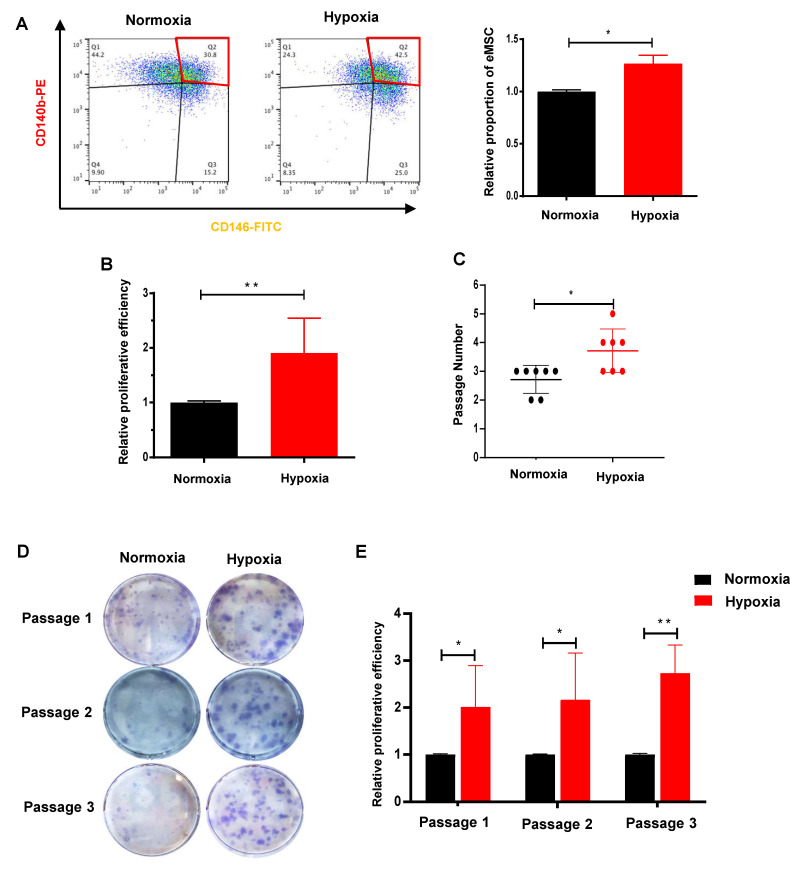
Hypoxia promotes eMSC proliferation and self-renewal. (**A**) Representative images and relative percentage of CD140b^+^CD146^+^ cells (red box) under normoxic (black bars) and hypoxic (red bars) conditions using flow cytometry (*n* = 4). (**B**) Relative proliferative ability (*n* = 6) and (**C**) self-renewal activity (*n* = 7) of eMSC in normoxia and hypoxia. (**D**) Representative figures showing the total colony formation of eMSC cultured under normoxia and hypoxia at different passage (*n* = 5–7). (**E**) Relative clonogenic efficiency of eMSC cultured under normoxia and hypoxia at different passage (*n* = 5–7). Results are presented as mean ± SD; * *p* < 0.05; ** *p* < 0.01. Abbreviation: eMSC, endometrial mesenchymal stem-like cells.

To investigate how O_2_ levels affect the properties of endometrial stem/progenitor cells, freshly isolated eMSC were cultured in normoxia (21% O_2_) and hypoxia (2% O_2_). It should be noted that the normoxic condition is approximately 5–7 times higher than the concentration of O_2_ in their natural niche [27]. The relative percentage of cells coexpressing CD140b and CD146 was significantly higher under the hypoxic condition when compared with the normoxic condition (Figure 3A, *n* = 4, *p* < 0.05). Similarly, the proliferation of eMSC increased remarkably after exposure to hypoxia (Figure 3B, *n* = 6, *p* < 0.01). Clonally derived eMSC in the hypoxic group underwent more rounds of self-renewal than the normoxic group (Figure 3C, *n* = 7, *p* < 0.05). There were also larger and densely packed colonies formed in the first three passages (Figure 3D,E, *n* = 5–7, *p* < 0.05 at passage 1 and passage 2, *p* < 0.01 at passage 3). These findings suggested that hypoxia enhanced the self-renewal capacity of eMSC and better maintained the eMSC phenotype in culture.

### 2.3. Hypoxia Activates the Notch Signaling in eMSC

**Figure 4:**
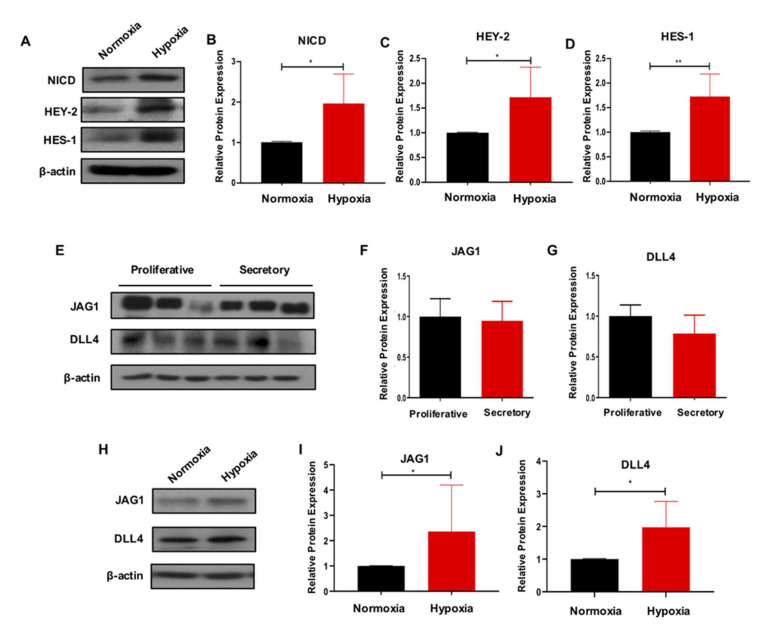
Hypoxia activates Notch signaling in eMSC and upregulates Notch ligands expression in myometrial cells. (**A**–**D**) Hypoxia activates Notch signaling in eMSC. Representative Western blotting images and quantitative analysis of Notch-related proteins expression in eMSC cultured under normoxia and hypoxia (*n* = 5–6). (**E**–**J**) Hypoxia upregulates Notch ligands in myometrial cells. (**E**–**G**) Representative Western blotting images and quantitative analysis of Notch ligand protein expression in myometrial cells from different menstrual phases (*n* = 3). (**H**–**J**) Representative Western blotting images and quantitative analysis of Notch ligands expression in myometrial cells cultured in normoxia and hypoxia (*n* = 5). Results are presented as mean ± SD; * *p* < 0.05. ** *p* < 0.01. Abbreviations: eMSC, endometrial mesenchymal stem-like cells.

To explore the molecular mechanism by which the hypoxic condition was affecting the phenotype of eMSC, we evaluated the activity of Notch signaling, which is widely reported to play a role in tissue homeostasis [20]. Under the hypoxic condition, the expression of Notch-related proteins NICD, HEY-2 and HES-1 was notably upregulated when compared to normoxia (Figure 4A–D, *n* = 6, *p* < 0.05 for NICD and HEY-2; *n* = 5, *p* < 0.01 for HES-1). The myometrial cells are candidate niche cells of eMSC [28]. Here, we found that the Notch ligands JAG1 and DLL4 were highly expressed in the myometrial cells, though their expression level did not change across the menstrual cycle (Figure 4E–G, *n* = 3). More importantly, Western blotting results demonstrated that hypoxia induced JAG1 and DLL4 expression in the myometrial cells (Figure 4H,J, *n* = 5, *p* < 0.05).

### 2.4. Hypoxia Requires Notch Signaling to Drive the Maintenance of eMSC

**Figure 5:**
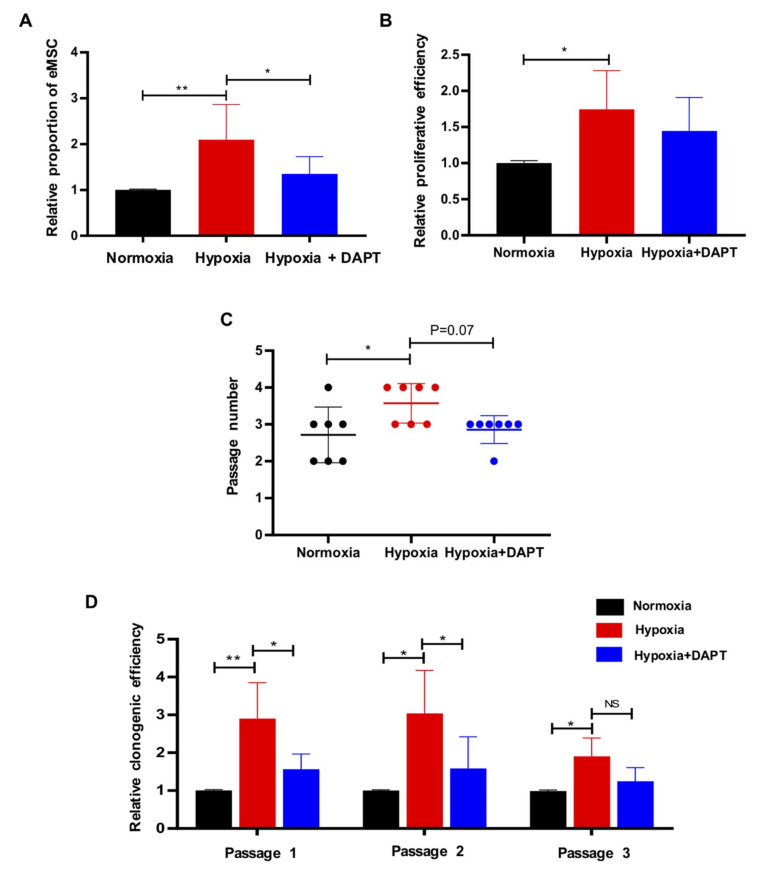
Hypoxia requires Notch signaling to drive the maintenance of eMSC. (**A**) Relative percentage of CD140b^+^CD146^+^ cells cultured under different conditions by flow cytometry (*n* = 6). (**B**) Relative proliferative ability of eMSC cultured under different conditions (*n* = 6). (**C**) The self-renewal activity of eMSC cultured under different conditions (*n* = 7). (**D**) Relative clonogenic efficiency of eMSC cultured under different conditions at different passages (*n* = 4–6). Results are presented as mean ± SD; * *p* < 0.05; ** *p* < 0.01. Abbreviation: eMSC, endometrial mesenchymal stem-like cells.

**Figure 6:**
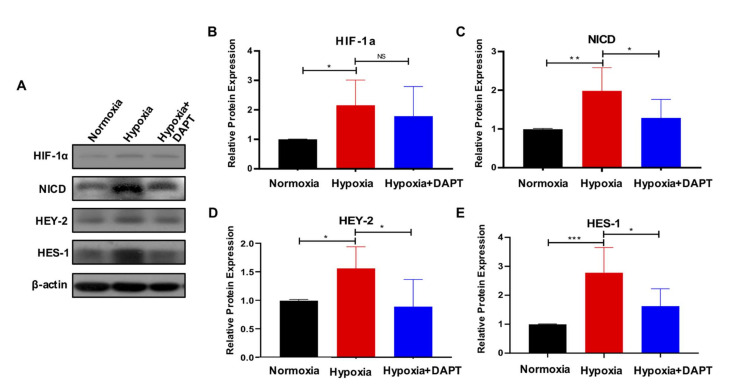
Upregulation of Notch activity in eMSC cultured under hypoxia is reversed by DAPT. (**A**) Representative Western blotting images of HIF-1α and Notch-related proteins expression in eMSC cultured under different conditions. (**B**–**E**) Relative quantitative analysis of HIF-1α and Notch-related proteins expression in eMSC cultured under different conditions (*n* = 6). Results are presented as mean ± SD; * *p* < 0.05; ** *p* < 0.01; *** *p* < 0.001. Abbreviation: eMSC, endometrial mesenchymal stem-like cells.

To test whether the hypoxia-mediated maintenance of eMSC required Notch signaling, eMSC were simultaneously treated with hypoxia and a γ-secretase inhibitor (DAPT), which inhibited the Notch receptor cleavage and thus prevented the release of NICD from the membrane [29]. Consistently, the proportion of CD140b^+^CD146^+^ cells was significantly higher in the hypoxic condition (Figure 5A, *n* = 6, *p* < 0.01) and DAPT nullified the stimulatory effect of hypoxia (Figure 5A, *n* = 6, *p* < 0.05). A decreasing trend in the proliferation of eMSC was observed when they were exposed to hypoxia in the presence of DAPT (Figure 5B, *n* = 6, *p* = 0.34) and underwent less rounds of self-renewal when compared to normoxia (Figure 5C, *n* = 7, *p* = 0.07). DAPT also abolished the positive regulating effect of hypoxia on the clonogenicity of eMSC at passage 1 and passage 2 (Figure 5D, *n* = 6, *p* < 0.05). Western blotting further confirmed that the activation of Notch signaling in eMSC under the hypoxic condition was reduced upon DAPT treatment (Figure 6A,C–E, *n* = 6, *p* < 0.05). As expected, hypoxia elevated the protein level of hypoxia-inducible factor-1α (HIF-1α) in eMSC (Figure 6A,B, *n* = 6, *p* < 0.05), while DAPT could not reduce the increase in HIF-1α expression (Figure 6A,B, *n* = 6, *p* = 0.64). Taken together, the results showed that hypoxia promoted the maintenance of eMSC via Notch signaling.

### 2.5. HIF-1a Is Essential for Hypoxia-Mediated Maintenance of eMSC by Activating Notch Signaling

**Figure 7:**
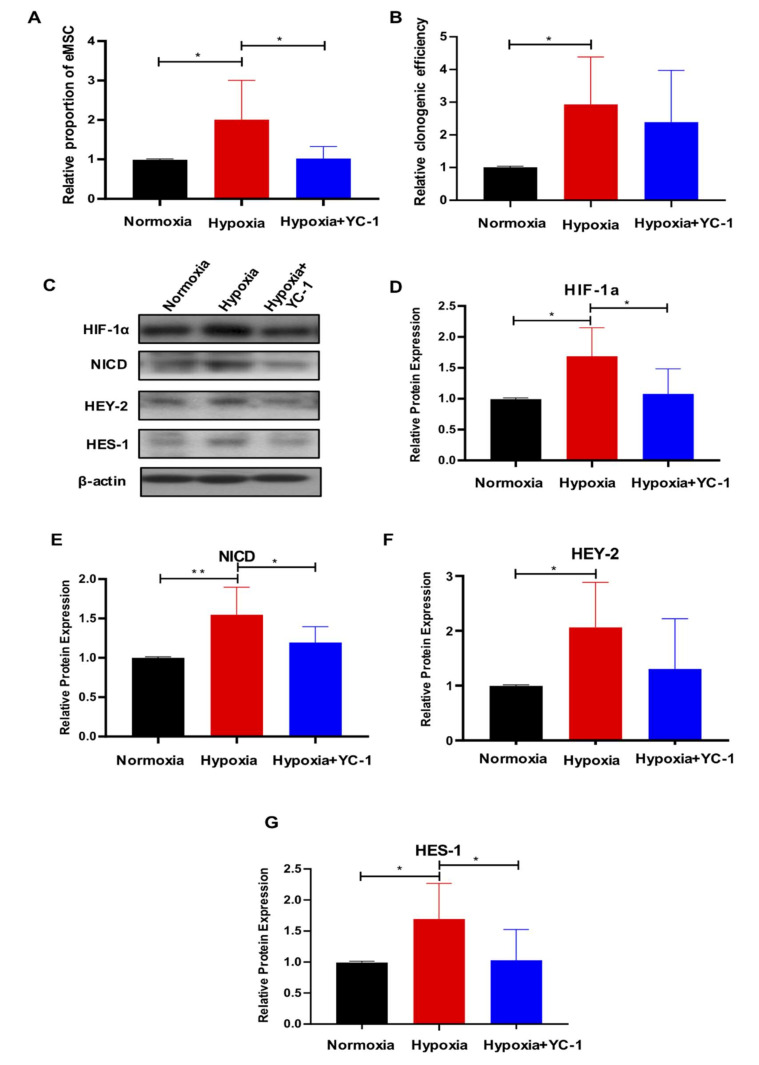
HIF-1α is essential for hypoxia-mediated maintenance of eMSC by activating Notch signaling. (**A**) Relative percentage of CD140b+CD146+ cells cultured under different conditions by flow cytometry (*n* = 6). (**B**) Relative clonogenic efficiency of eMSC cultured under different conditions (*n* = 6). (**C**) Representative Western blotting images of HIF-1α and Notch-related proteins expression in eMSC cultured under different conditions. (**D**–**G**) Relative quantitative analysis of HIF-1α and Notch-related proteins expression in eMSC cultured under different conditions (*n* = 5–6). Results are presented as mean ± SD; * *p* < 0.05; ** *p* < 0.01. Abbreviation: eMSC, endometrial mesenchymal stem-like cells.

It has been reported that Notch signaling and HIF-1α undergoes crosstalk in hypoxic cells [30]. Therefore, we evaluated the activity of Notch signaling in eMSC when HIF-1α was pharmacologically inhibited in hypoxic conditions. Flow cytometry results showed that the presence of the HIF-1α inhibitor YC-1 abrogated the hypoxia-induced effect on the proportion of CD140b^+^CD146^+^ cells (Figure 7A, *n* = 6, *p* < 0.05), and there was a declined trend on the colony-formation ability of eMSC (Figure 7B, *n* = 6, *p* = 0.54). Upon exposure to hypoxia, the expression of HIF-1α and Notch-related proteins NICD, HEY-2 and HES-1 dramatically increased (Figure 7C–G, *n* = 6, *p* < 0.05 for HIF-1α, HEY-2 and HES-1; *n* = 6, *p* < 0.01 for NICD). Treatment with YC-1 significantly reduced the protein expression of HIF-1α, NICD and HES-1 (Figure 7C–E,G, *n* = 6, *p* < 0.05). These data indicated that hypoxia enhanced the expression of HIF-1α, which resulted in the activation of Notch signaling and the maintenance of eMSC.

## 3. Discussion

In this study, the cellular and histological events occurring in the artificially decidualized mouse endometrium recapitulate the events that occur in the human endometrium at menstruation and early regeneration. We detected a portion of the LRSC experienced a hypoxic microenvironment during the dynamic remodeling events in the mouse endometrium. Hypoxia promoted the self-renewal and clonogenicity of eMSC in vitro and activated the underlying molecular Notch signaling pathway. Furthermore, HIF-1α played a critical role in the hypoxia-mediated maintenance of eMSC through the activation of Notch signaling. To our knowledge, this is the first study investigating the potential role of hypoxia and Notch signaling on endometrial stem/progenitor cells both in vitro and in vivo.

It has been hypothesized that endometrial breakdown during menstruation in humans was initiated by tissue hypoxia resulting from vasoconstriction of the spiral arterioles [13]. Recently, the existence and function of hypoxia in the endometrium has been challenged by several groups [17,31,32]. Previous studies revealed that physiological hypoxia could be observed in mouse endometrium of simulated menstruation by applying the hypoxyprobe pimonidazole [15,16,33]. Consistent with their findings, we detected a transient but intense hypoxia episode in the decidualized endometrium of pseudopregnant mice displaying dynamic remodeling events. The hypoxia gradually reduced and eventually disappeared when the mouse endometrial repair was completed.

Physiologically, stem cells of various tissues are located in hypoxic niches in vivo and their functions are greatly regulated by local oxygen concentrations [3]. For example, a low oxygen concentration has been detected in the tissue niches where MSC reside in vivo [34]. Here, we provided direct evidence that a small population of LRSC experienced hypoxia when endometrial breakdown occurred. Additionally, we found that a significant proportion of these hypoxic LRSC underwent proliferation. A recent study revealed that the prevention of hypoxia in the mouse model of simulated menstruation significantly delayed endometrial repair [15]. Therefore, combining their results with the present study, we propose that hypoxia activates endometrial stem/progenitor cells and contributes to endometrial regeneration after breakdown.

Hypoxia mediates self-renewal and other characteristics of various somatic stem cells [9,35,36]. It has been reported that hypoxia maintained the neural stem cells in an undifferentiated state and increased their proliferation [23]. Similarly, our results showed that hypoxia significantly promoted the self-renewal ability and colony formation potential of eMSC. These results are in agreement with findings of other researchers who investigated the effects of hypoxia on MSC [37,38,39]. Their results suggest that hypoxic conditions increase the stemness, cell proliferation and multipotency of MSC in culture [34]. To date, the accurate determination of human endometrial hypoxia has not been possible. Reavy and coworkers applied the MRI technique to measure endometrial hypoxia. By quantifying T2*, a technique to evaluate tissue oxygenation status, they reported that T2* values declined significantly during menstruation compared to the nonmenstrual phases of the cycle. For oxygen tension in the myometrium, the endometrial–myometrial junction was reported to have a lower T2* value than the outer myometrium. This indirect evidence further suggests a hypoxic microenvironment exists in the basal layer of the human endometrium, where stem cells are located [40].

Whether hypoxia activates the Notch signaling is controversial [8,22,41]. For example, hypoxia exerts its effects on satellite cells by activating Notch signaling [8]. Conversely, Itoh et al. found that hypoxia suppressed the self-renewal of leukemia cells by inhibiting Notch signaling [41]. Here, our results not only supported the notion that hypoxia induced the upregulation of Notch activity in eMSC, but further demonstrated that Notch signaling was required to meditate the effect of hypoxia on eMSC activities, as the pharmacological inhibition of Notch signaling reversed the advantageous outcomes. This is in accordance with the work of Moriyama et al., which demonstrated that Notch signaling was essential for the maintenance of MSC in the hypoxic condition [22]. We also found that hypoxia induced the expression of JAG1 and DLL4 in myometrial cells. Therefore, it would be interesting to study whether hypoxia could promote the intercellular communication via Notch signaling.

In the current study, we also investigated the molecular mechanism involved in the activation of Notch signaling in eMSC in a hypoxic condition. Emerging evidence suggests that HIF-1α, a key factor associated with hypoxia and functions as a link between the hypoxic cue and the Notch response in hypoxic cells [42,43,44]. Gustafsson et al. revealed the direct interaction between HIF-1α and NICD that maintained neural stem cells in an undifferentiated state under a low oxygen condition [44]. Similarly, Qiang et al. reported that HIF-1α might play an important role in the hypoxia-mediated maintenance of glioblastoma stem cells, partly via its interaction with NICD [21]. Here, our results showed that hypoxia not only abundantly increased the protein expression of HIF-1α, but also upregulated the activity of Notch signaling in eMSC. Pharmacological inhibition of HIF-1α with YC-1 abolished this hypoxia-induced Notch activation in eMSC. Nevertheless, the suppression of Notch signaling by DAPT could not reverse the upregulation of HIF-1α in eMSC under hypoxia. These data suggest HIF-1α is the upstream of Notch signaling and that hypoxia regulates the properties of eMSC by activating Notch signaling in a HIF-1α-dependent manner. Recently, several studies explored the function of HIF-1α in endometrial repair during menstruation using mouse menstrual-like models [15,16]. It was reported that the genetic or pharmacological reduction of endometrial HIF-1α significantly delayed the endometrial repair [15]. In our murine model, a high percentage of hypoxic LRSC colocalized with Notch1 during endometrial breakdown and regeneration. Whether a crosstalk between HIF-1α and Notch1 exists during these events needs further investigation.

## 4. Materials and Methods

### 4.1. Animal and Housing Condition

Mice were provided by the Center of Comparative Medicine Research at the University of Hong Kong. All experimental procedures performed in this study were approved by the Committee on Use of Live Animals in Teaching and Research, the University of Hong Kong, Hong Kong. The mice were kept under standard conditions with a light/dark cycle of 12 h/12 h and free access to food and water.

### 4.2. Animal Study Design

The experimental setup is shown in Supplementary Figure S1. Day 19 prepubertal C57BL/6J female mice were labeled with BrdU according to our previous study [18]. After a 6-week chase, the standard protocol to induce endometrial breakdown and repair was performed [19]. The BrdU^+^ cells after a 6-week chase were referred as LRSC in the present menstrual-like model. In brief, female mice were mated with vasectomized >6-week-old C57BL/6J male mice (day 0). Pseudopregnant female mice were identified by the presence of a vaginal plug on the next day (day 1). On day 4 of pseudopregnancy, 30 µL of sesame oil was injected into the left uterine horn to induce decidualization, while the right horn was not treated as control. The mice were euthanized, and their uteri were harvested on day 4 of pseudopregnancy (before decidualization), decidualization (day 7), breakdown (day 9), early repair (day 10) and late repair (day 12). Mice received an intraperitoneal injection of pimonidazole (60 mg/kg, 4.3.11.3 mouse MAb, Hypoxyprobe, Burlington, MA, USA) 1.5 h before being euthanized. The harvested uteri were fixed with 4% paraformaldehyde overnight and processed into paraffin blocks.

### 4.3. Immunohistochemistry of Pimonidazole

Immunohistochemical analysis of Hypoxyprobe (pimonidazole) were conducted according to the manufacturer’s instructions. The paraffin sections (5 µm) were deparaffinized and dehydrated. Antigen retrieval was performed using antigen retrieval buffer (Dako, Hamburg, Germany) in a microwave oven, followed by incubation with 3% H_2_O_2_ for 10 min at room temperature. To reduce nonspecific staining, the sections were blocked with 5% BSA for 30 min at 37 °C, then incubated with pimonidazole primary antibody (1:50 dilution, mouse Mab, Burlington, MA, USA) at 4 °C overnight. The following day, the sections were incubated with polyclonal goat anti-mouse biotinylated secondary antibodies (1:200 dilution, Dako, Hamburg, Germany) for 60 min, and Vectastain ABC Standard Kit reagents (Vector Laboratories, Burlington, MA, USA) for another 30 min. A diaminobenzidine (DAB) kit (Dako, Hamburg, Germany) was applied for color development and monitored under a Zeiss Axioskop II microscope (Carl Zeiss, Munich, Germany). The sections were counterstained with the Mayer’s hematoxylin for 1 min, dehydrated and mounted using aqueous mounting medium (Dako). Images were captured using a Photometrics CoolSNAP digital camera (Roper Scientific, Trenton, NJ, USA). All incubations were performed at room temperature and washes with PBS were conducted between each step.

### 4.4. Dual and Triple Immunofluorescence Staining

Paraffin sections were dewaxed and underwent antigen retrieval, followed by denaturation with 0.1 *n* HCl for 45 min. The sections were then quenched with 3% hydrogen peroxide for 10 min and blocked with 5% BSA/PBS for 1 h. For dual immunofluorescence staining, the two primary antibodies (Supplementary Table S2) were coincubated at 4 °C overnight. The slides were then incubated with the corresponding secondary antibodies (Supplementary Table S3) at 37 °C for 1 h. For triple staining, the anti-BrdU antibody staining was conducted first, followed by incubation with the other two primary antibodies at 4 °C overnight and subsequently the corresponding secondary antibodies on the next day. The slides were stained with DAPI (Thermo Scientific, Waltham, MA, USA) and mounted with a fluorescence mounting medium (Dako). Multispectral fluorescence images were captured by a Carl Zeiss LSM 800 inverted confocal microscope and the Zeiss LSM ZEN 2019 software (Carl Zeiss, Munich, Germany) at the CPOS, the University of Hong Kong.

### 4.5. Human Endometrial Tissues

Human endometrial samples (*n* = 21) were obtained from women with regular menstrual cycles, aged 41–52 years old, undergoing hysterectomy for benign nonpathological conditions. Women recruited in this study had not received any hormone treatment for at least three months. The phase of the menstrual cycle was categorized into proliferative (*n* = 11) and secretory (*n* = 10) by experienced histopathologists based on hematoxylin–eosin-stained endometrial sections.

### 4.6. Isolation of Endometrial Stromal Cells

The isolation of single endometrial stromal cells was performed according to our previous study [28]. In brief, endometrial tissue was minced into small pieces and digested with PBS containing collagenase type III (0.3 mg/mL, Worthington Biochemical Corporation, NJ, USA) and deoxyribonuclease type I (40 μg/mL, Worthington Biochemical Corporation, NJ, USA) at 37 °C for 1 h. After two rounds of digestion, Ficoll-Paque (GE Healthcare, Uppsala, Sweden) centrifugation and anti-CD45 antibody coated Dynabeads (Invitrogen, Waltham, MA, USA) were sequentially used to remove the red blood cells and the leukocytes, respectively. Purified stromal cells were then separated from epithelial cells using anti-CD326 (EpCAM) antibody-coated microbeads (Miltenyi Biotech, San Diego, CA, USA). Next, freshly isolated stromal cells were seeded into 100 mm dishes coated with fibronectin (1 mg/mL, Life Technologies, Carlsbad, CA, USA) and cultured in growth medium (GM) containing 10% FBS (Invitrogen, Waltham, MA, USA), 1% L-glutamine (Invitrogen, Waltham, MA, USA) and 1% penicillin-streptomycin (Invitrogen, Waltham, MA, USA) in DMEM/F-12 (Sigma-Aldrich, St Louis, MA, USA). Stromal cells were cultured in a humidified carbon dioxide incubator at 37 °C. The medium was changed every 7 days until the cells reached 80% confluence.

### 4.7. Magnetic Bead Selection for Endometrial Mesenchymal Stem-like Cells

Two successive magnetic bead selections were carried out to isolate the CD140b^+^ and CD146^+^ cells (eMSC) [28]. Firstly, stromal cells were incubated with phycoerythrin (PE)-conjugated anti-CD140b antibody (R&D Systems, Minneapolis, MN, USA) for 45 min at 4 °C followed by another 15 min incubation with anti-mouse IgG1 magnetic microbeads (Miltenyi Biotech, San Diego, CA, USA). The cell suspensions were then loaded onto MS columns (Miltenyi Biotech, San Diego, CA, USA) with a magnetic field to separate the CD140b^+^ cells, which were cultured for 7–10 days to allow degradation of the microbeads during cell expansion. The cells were then trypsinized and incubated with anti-CD146 antibody-coated microbeads (Miltenyi Biotech, San Diego, CA, USA) for 15 min at 4 °C to obtain the CD140b^+^CD146^+^ cells for experimentation [28]. Normal humidified cell incubator with 5% CO_2_ was used for normoxic cultures (21% O2). For hypoxic cultures (2% O_2_), the cells were placed into a sealed chamber (Thermo Fisher Scientific, Waltham, MA, USA), which was flushed with a gas mixture of 2% O_2_, 5% CO_2_, and 93% N_2_ (*v/v*) at 37 °C.

### 4.8. Preparation of Human Endometrial Myometrial Cells

Myometrial samples (*n* = 12, proliferative phase: *n* = 7, secretory phase: *n* = 5) were collected from women, aged 43–48 years old (mean age: 45.4 years), who underwent hysterectomy for benign nonendometrial pathologies. Isolation of myometrial cells was carried out as described [28]. Myometrial tissue was digested in 5 mL PBS containing collagenase type III (300 μg/mL) and deoxyribonuclease type I (40 μg/mL) for 3 h at 37 °C. After digestion, single cell suspensions were filtered through 100 μm sieves (BD Bioscience, San Jose, CA, USA) and seeded onto 100 mm dishes with GM in a humidified incubator at 37 °C. The myometrial cells were trypsinized and passaged when they reached ~80% confluence. Myometrial cells at passage 2 to 6 were used in this study.

### 4.9. Preparation of Notch Signaling Inhibitor DAPT

N-[N-(3,5-difluorophenacetyl- l- alanyl)]-(S)-phenylglycine t-butyl ester (DAPT; R&D Systems, Minneapolis, MN, USA) was dissolved in DMSO at a concentration of 10 mM and stored at −20 °C. Freshly isolated eMSC were seeded onto fibronectin-coated 6-well plates at clonal density (400 cells/well). Cells were cultured under hypoxic conditions and treated with DAPT at a concentration of 1.25 µM for 15 days. The GM was changed every 3 days. GM containing DMSO served as control.

### 4.10. Preparation of HIF-1α Inhibitor YC-1

YC-1 (Sigma-Aldrich, St Louis, USA) was dissolved in DMSO at a concentration of 10 mM and stored at-20 °C. Freshly isolated eMSC were seeded onto fibronectin-coated 6-well plates at clonal density (400 cells/well). The cells were cultured in hypoxic conditions and treated with YC-1 at a concentration of 10 µM for 15 days. The GM was changed every 3 days. GM containing DMSO served as control.

### 4.11. Flow Cytometry

Multicolor flow cytometry was applied to analyze the coexpression of CD140b and CD146 on endometrial stromal cells. The cells were incubated with PE-conjugated anti-CD140b antibody (2.5 μg/mL, PR7212 clone, Mouse IgG1, R&D Systems, Minneapolis, MN, USA) and FITC-conjugated anti-CD146 antibodies (5 μg/mL, OJ79c clone, mouse IgG1; ThermoFisher Scientific, Waltham, MA, USA) in the dark for 45 min at 4 °C. Fluorescent minus one (FMO) control was included for each antibody. Following the final washing step, the labeled cells were analyzed by a CytoFlex™ flow cytometer (Beckman Coulter, CA, USA). The cells were selected with electronic gating according to the forward and the side scatter profiles. Data were analyzed by the FlowJo Software (Tree Star Inc, Ashland, OR, USA).

### 4.12. In Vitro Colony Forming Assay

Freshly isolated eMSC were seeded onto 6-well plates (400 cells/well) and cultured under different conditions for 15 days to form colonies. Medium was changed every 3 days. The cloning efficiency was evaluated by the number of colonies divided by the number of seeded cells multiplied by 100.

### 4.13. In Vitro Serial Cloning

Individual colonies were trypsinized using cloning rings to evaluate the self-renewal ability of cells cultured in different conditions. Two individual colonies per sample were used for serial cloning. The cell number of each CFUs was counted and the cells were re-seed onto 6-well plates (400 cells/well) to form colonies. Three conditions were set up: (1) normoxia, (2) hypoxia and (3) hypoxia in the presence of DAPT. The process continued until the cells were no longer able to form CFUs.

### 4.14. Cell Proliferation Assay

The CyQUANT™ NF Cell Proliferation kit (Thermo Scientific, Waltham, MA, USA) was used to determine the proliferative ability of eMSC. In brief, eMSC (1 × 10^3^ cells/well) were seeded into 96-well plates and cultured under different conditions for 3 days. Cells treated with DMSO were used as control. After washing with PBS, 100 μL of dye binding solution was added to each well and cultured for 1 h at 37 °C. Fluorescence intensity was measured by a fluorescence microplate reader with excitation at 485 nm and emission at 530 nm.

### 4.15. Western Blotting Analysis

Cultured eMSC were lysed in cell lysis buffer (Ambion, Grandisland, NY, USA) in the presence of protease inhibitors. Then, 5 μg of the denatured protein samples were separated on 10% SDS-PAGE and transferred to polyvinylidene difluoride membranes (Immobilon™-P, Milllipore). After blocking with 5% skim milk for 1 h at room temperature, the membranes were incubated with appropriate primary antibodies overnight at 4 °C followed by horseradish peroxidase-conjugated secondary antibodies for 1 h at room temperature (Supplementary Table S4). The protein expression was detected by the Western Bright ECL Kit (Advansta, CA, USA). The relative expression of the target proteins was normalized to housekeeping protein β-actin.

### 4.16. Statistical Analysis

Data were analyzed using the GraphPad PRISM software (version 8.00; GraphPad Software Inc., San Diego, CA, USA). Distribution normality was tested using the Shapiro–Wilk test. Differences between two groups were analyzed using the Mann–Whitney U-test for nonparametric data and the two-tailed unpaired Student’s t-test for parametric data. One-way ANOVA followed by Tukey’s test or the Kruskal–Wallis test, followed by Dunn’s test, were used for multiple group comparison. Data are represented as mean ± SD. A difference with *p*-value of <0.05 is considered as significant.

## 5. Conclusions

Using the LRC technique, we demonstrated that a population of LRSC (candidate stromal stem/progenitor cells) encountered a hypoxic condition during endometrial breakdown and early regeneration using a mouse menstrual-like model. In this study, we also found that hypoxia promoted the self-renewal and clonogenicity of eMSC in vitro and that HIF-1a was essential for the hypoxia-induced maintenance of eMSC by activating Notch signaling.

## Data Availability

Not applicable.

## Supplementary Materials

The following supporting information can be downloaded at: https://www.mdpi.com/article/10.3390/ijms23094613/s1.
