# Supplementary material for: Hypoxia Regulates the Self-Renewal of Endometrial Mesenchymal Stromal/Stem-like Cells via Notch Signaling
Source: Int J Mol Sci. 2022 Apr 21;23(9):4613. doi: 10.3390/ijms23094613 (PMC9104239; doi:10.3390/ijms23094613)

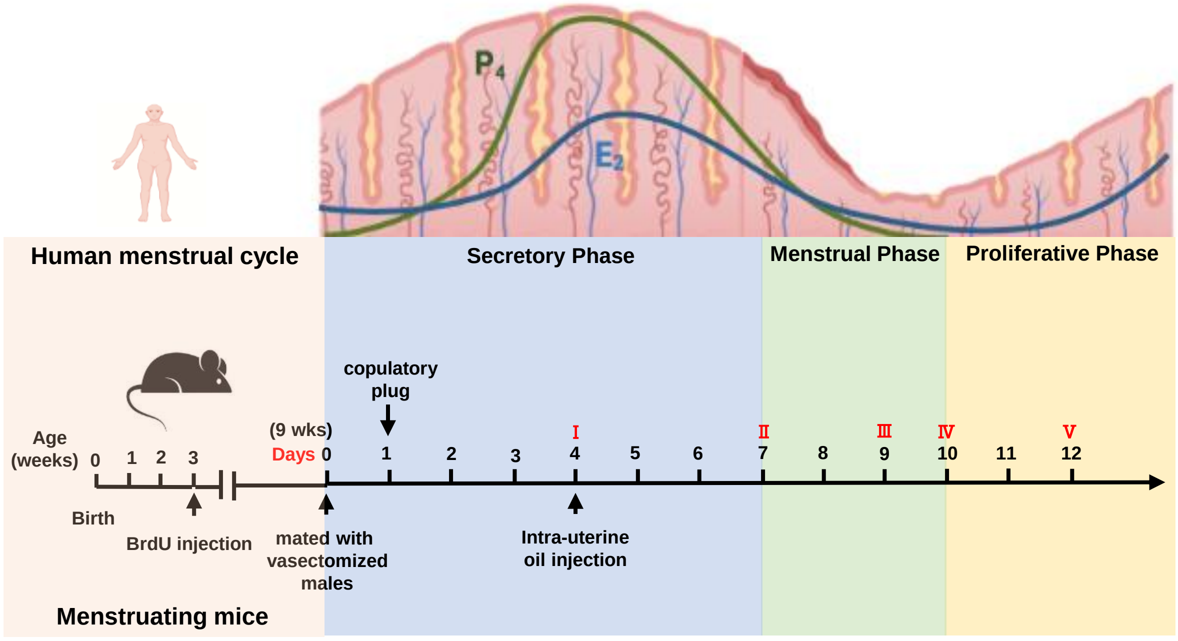

Figure S1. Schematic comparison of human menstrual cycle and mouse menstrual-like model. Upper panel depicts a schematic of the human endometrium and its changes through the menstrual cycle consequent upon circulating concentrations of estradiol (E_2) and progesterone (P_4). Lower panel depicts a schematic protocol of the mouse menstrual-like model. Prepubertal (day 19) C57BL/6J female mice were pulse labeled with BrdU (50 mg/g of body weight) twice daily for 4 days. After chasing for 5-6 weeks, female mice were mated with vasectomized male mice (day 0). Pseudo pregnant female mice were identified by vaginal plugs on the next day (day 1). On day 4, endometrial decidualization was induced by intrauterine injection of sesame oil. Tissue was collected at day 4 (before decidualization, denoted by Ⅰ), day 7 (decidualization, denoted by Ⅱ), day 9 (breakdown, denoted by Ⅲ), day 10 (early repair, as denoted by Ⅳ), and day 12 (late repair, denoted by Ⅴ).

Figure S2. Uncropped scans of western blots (Figure 4A). The representative western blotting images is highlighted with the red boxes. N: normoxia; H: hypoxia

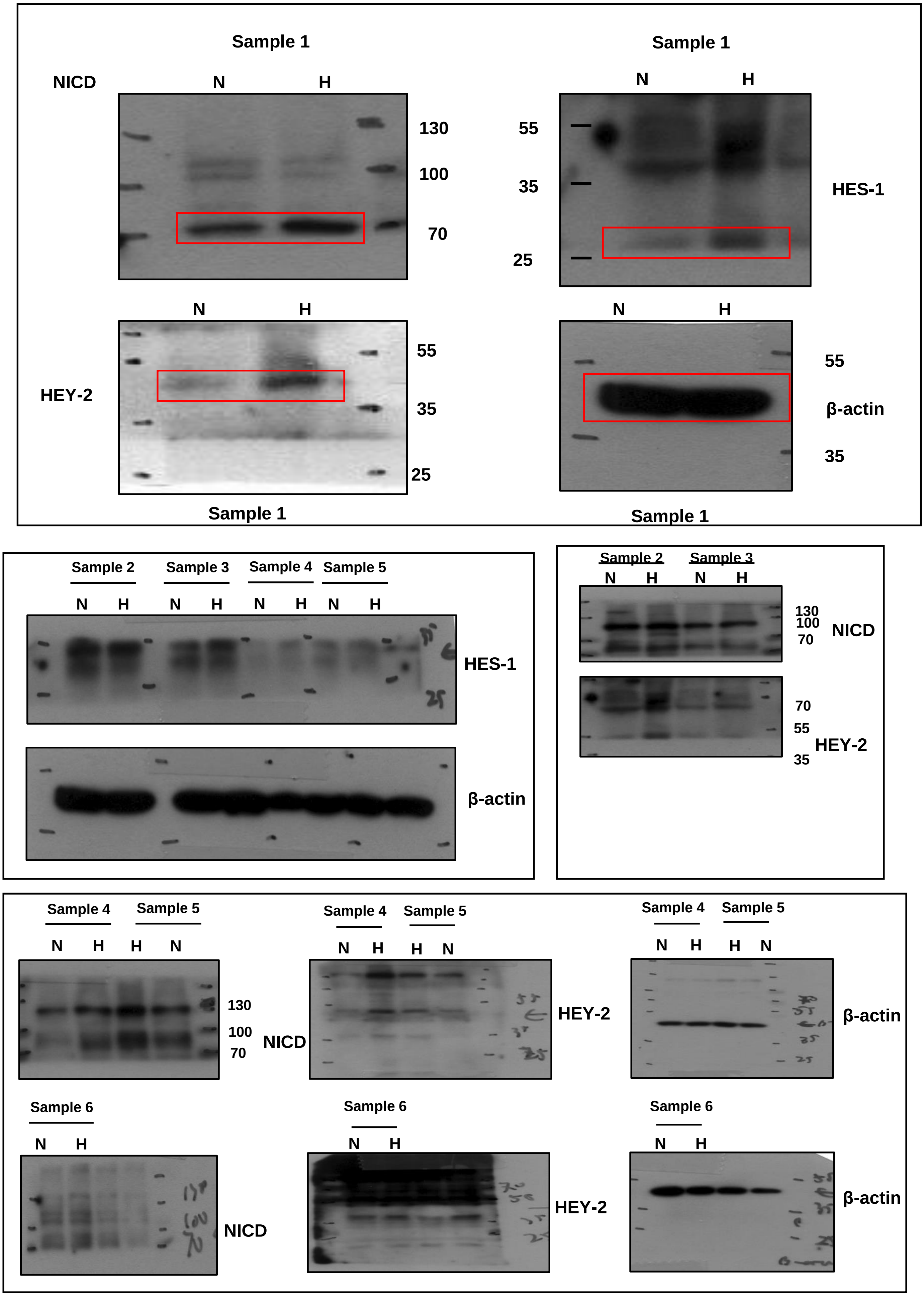

Figure S3. Uncropped scans of western blots (Figure 4E). The representative western blotting images is highlighted with the red boxes.

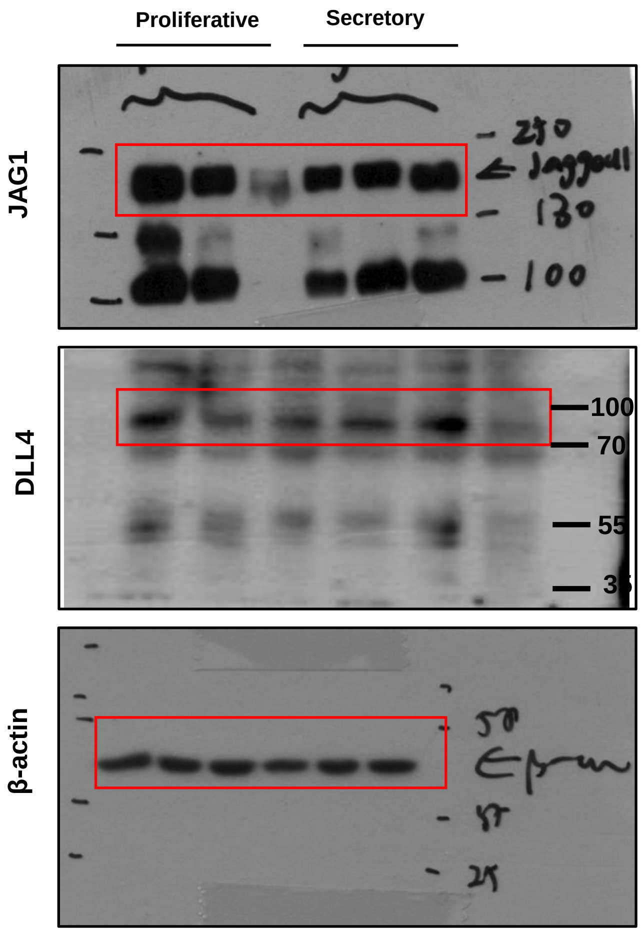

Figure S4. Uncropped scans of western blots (Figure 4H). The representative western blotting images is highlighted with the red boxes. N: normoxia; H: hypoxia

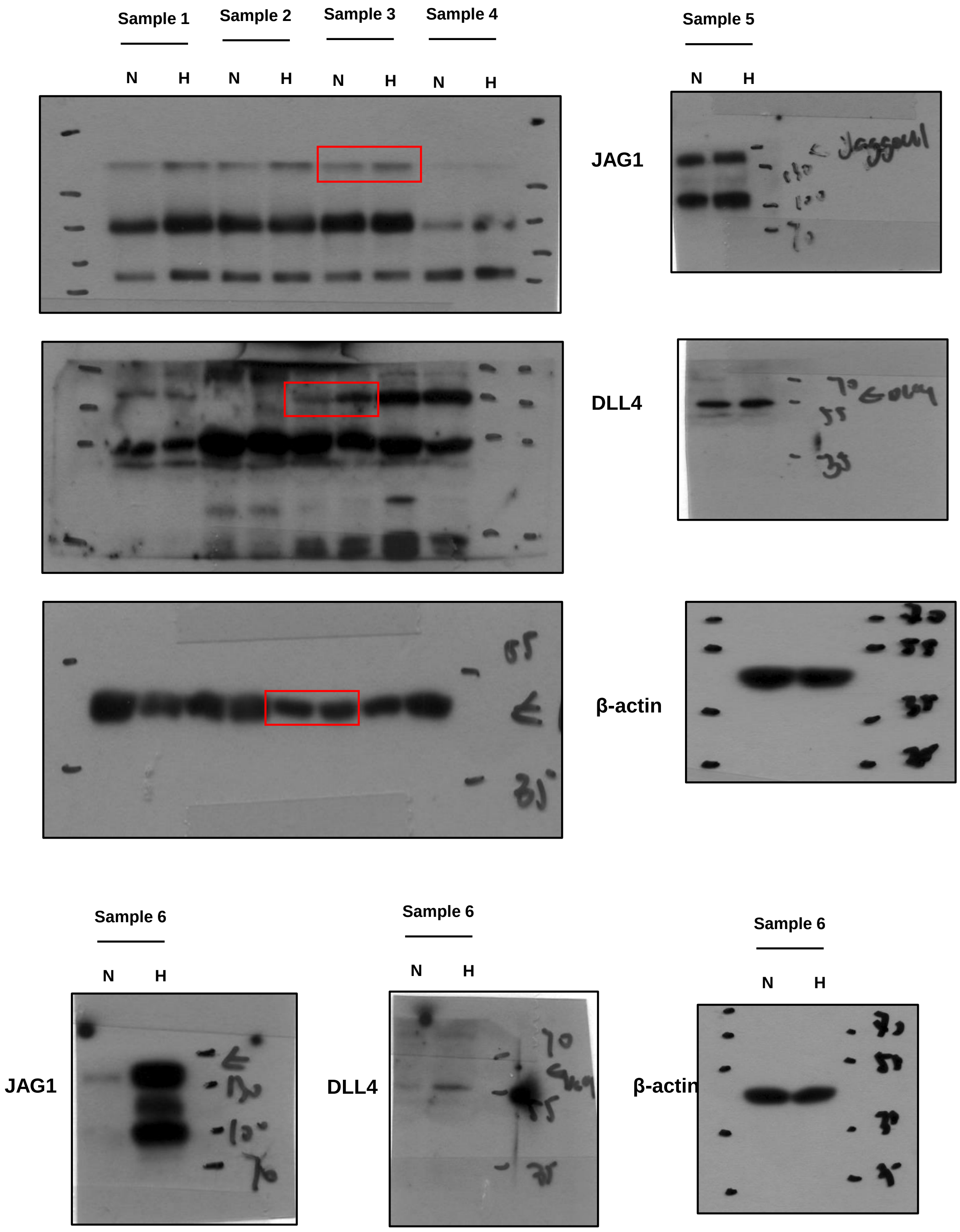

Figure S5. Uncropped scans of western blots (Figure 6A). The representative western blotting images is highlighted with the red boxes. N: normoxia; H: hypoxia; D: hypoxia+DAPT

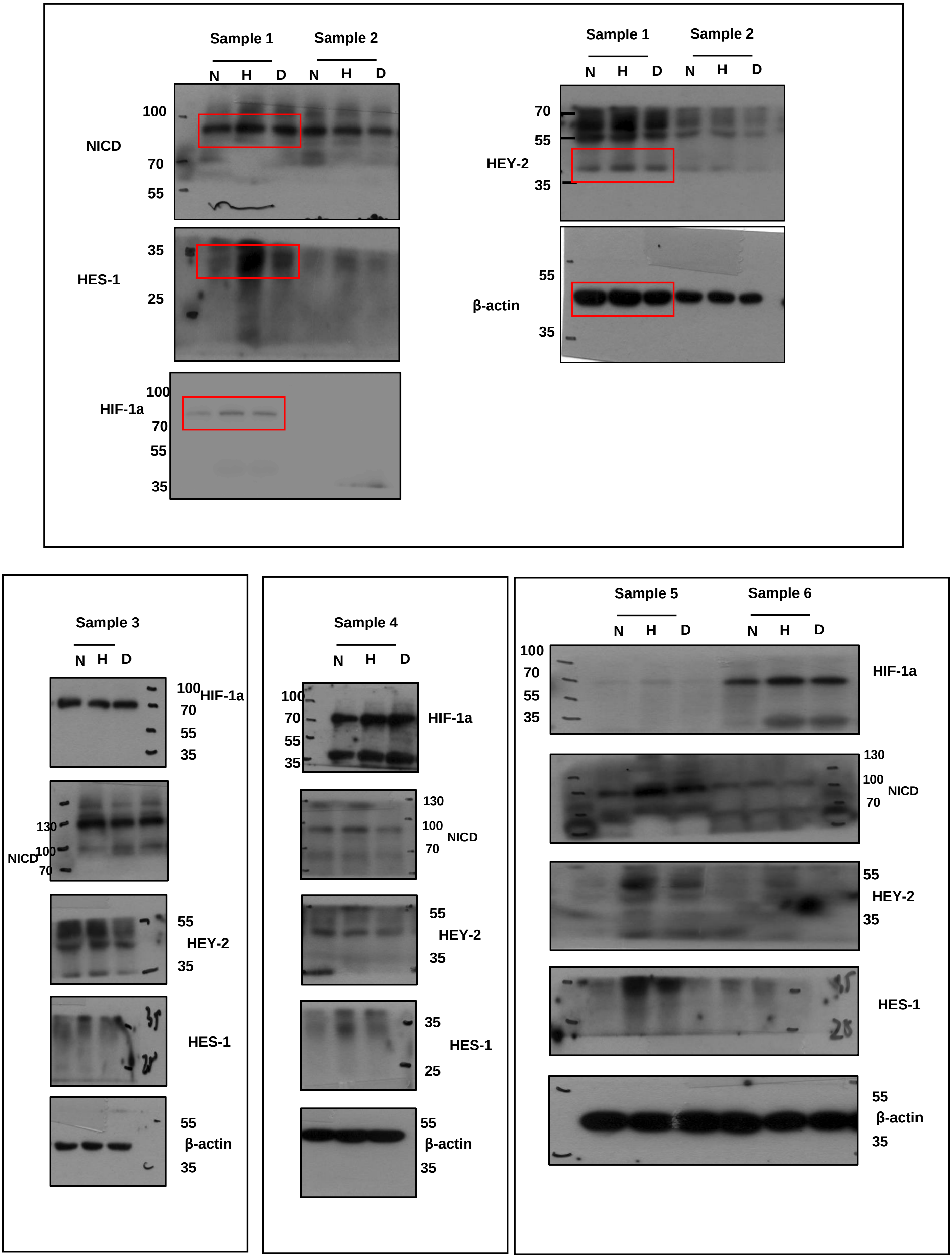

Figure S6. Uncropped scans of western blots (Figure 7C). The representative western blotting images is highlighted with the red boxes. N: normoxia; H: hypoxia; Y: hypoxia+YC-1

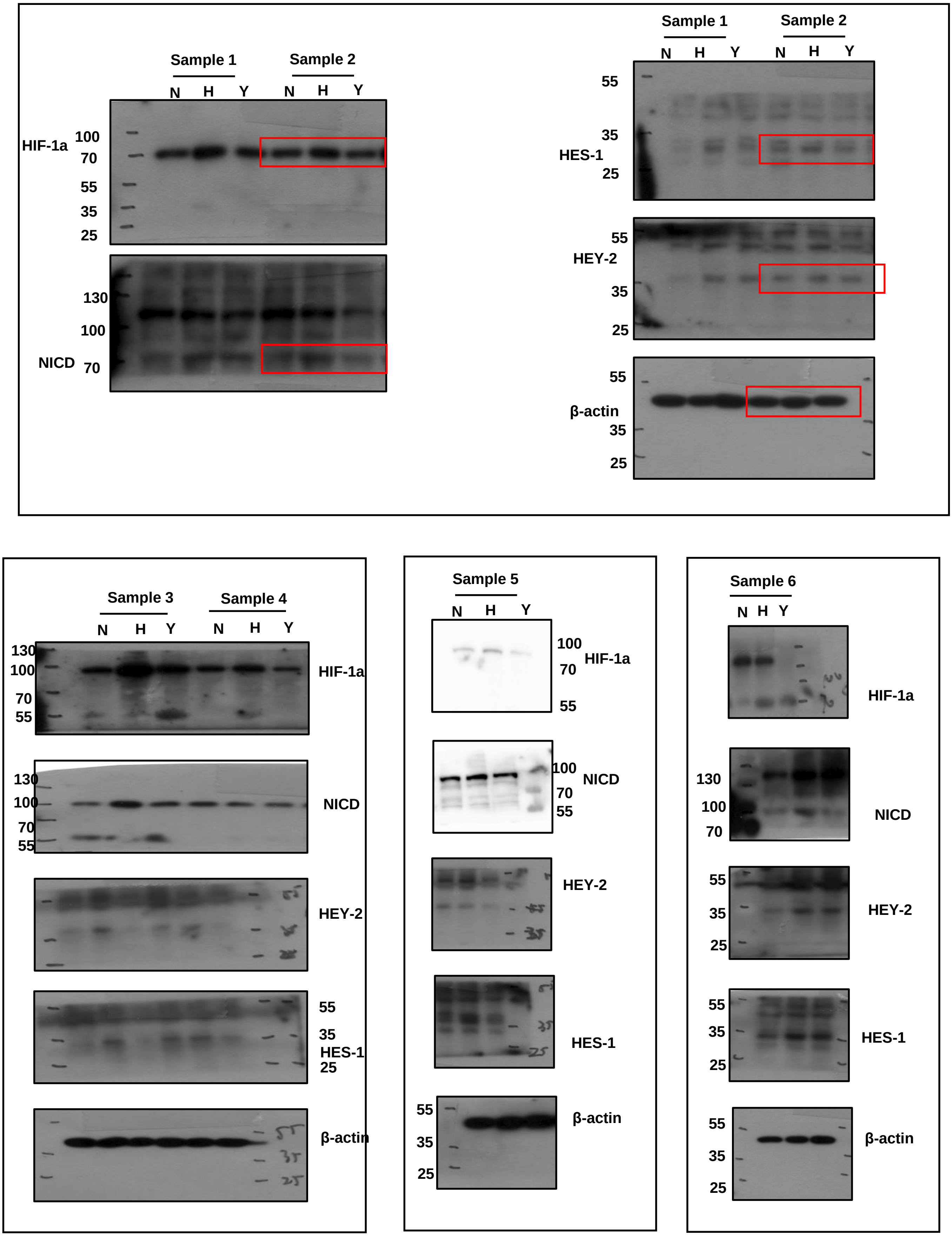

Supplement: Supplementary file 1 [file ijms-23-04613-s001.zip › ijms-1654409-supplementary.pdf]
